# Diffuse large B‐cell lymphoma mimicking cardiac amyloidosis

**DOI:** 10.1002/ccr3.948

**Published:** 2017-04-17

**Authors:** Lawrence Lau, Viktoriya Mozolevska, Iain D. C. Kirkpatrick, Davinder S. Jassal, Roopesh Kansara

**Affiliations:** ^1^ Section of CardiologyDepartment of Internal MedicineUniversity of ManitobaWinnipegManitobaCanada; ^2^ Department of RadiologyUniversity of ManitobaWinnipegManitobaCanada; ^3^ Section of Hematology and Medical OncologyDepartment of Internal MedicineUniversity of ManitobaWinnipegManitobaCanada

**Keywords:** Cardiac amyloidosis, cardiac imaging, heart failure, malignancy, cardiac lymphoma

## Abstract

Our case highlights that cardiac lymphoma may mimic amyloid infiltration of the myocardium on cardiac magnetic resonance (CMR), and is a particularly challenging diagnosis in the setting of transformed Waldenström's macroglobulinemia. Heightened suspicion and early diagnosis of cardiac lymphoma is paramount as chemotherapy has been demonstrated to portent an increased survival rate.

## Clinical Case

**Figure 1:**
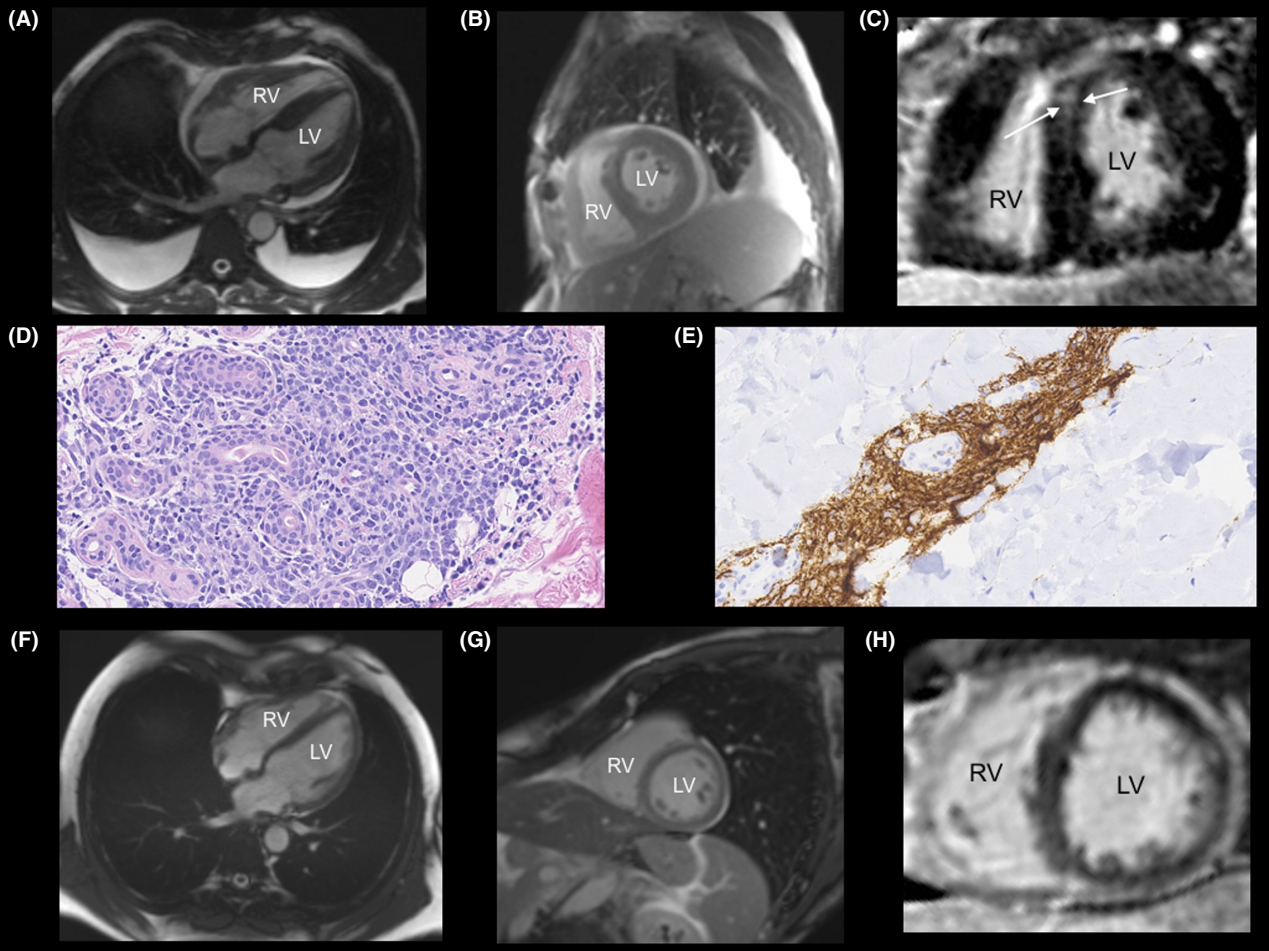
(A–B) Balanced steady‐state free precession (b‐SSFP) CMR imaging in (A) four‐chamber and (B) short‐axis views demonstrating biventricular hypertrophy, bilateral pleural effusions, and a small pericardial effusion. (C) Phase‐sensitive inversion recovery (PSIR) T1‐weighted gradient echo short‐axis image demonstrating mid‐myocardial DE (arrows). (D–E) Fat pad biopsy demonstrating atypical periadnexal lymphoid infiltrates (C) and staining positive for CD20 confirming DLBCL (D). (F–G) Repeat b‐SSFP CMR after 6 months of chemotherapy in (E) four‐chamber and (F) short‐axis views, demonstrating complete resolution of structural abnormalities. (H) Repeat PSIR T1‐weighted short‐axis image after chemotherapy demonstrating resolution of the DE within the myocardium.

A previously healthy 62‐year‐old man was admitted to the intensive care unit with acute heart failure and pancytopenia. Serum protein electrophoresis demonstrated an elevated IgM paraprotein level of 31 g/L. Bone marrow biopsy confirmed mature B‐cell lymphocytosis with an immunophenotype of CD20+, CD10−, CD5− and a focal increase in CD138. A diagnosis of Waldenström's macroglobulinemia was established. His presentation was complicated by markedly elevated levels of serum calcium and lactate dehydrogenase, suggesting transformation of his hematological disorder. Cardiac magnetic resonance (CMR) imaging revealed biventricular hypertrophy with patchy delayed enhancement (DE) throughout the left ventricular myocardium, which was highly suspicious of cardiac amyloidosis (Fig. 1A–C). A fat pad biopsy was negative for Congo red staining but confirmed CD20+ and CD10‐ diffuse large B‐cell lymphoma (DLBCL) (Fig. 1D and E). Promptly after the diagnosis of DLBCL, he was initiated on chemotherapy consisting of rituximab, cyclophosphamide, etoposide, vincristine, and prednisone. He received a total of six cycles of chemotherapy over the course of 6 months. End‐of‐therapy monoclonal immunoglobulin concentration declined from 31 to 5 g/L, and positron emission tomography confirmed no residual active metabolic disease. Repeat CMR imaging demonstrated complete resolution of both the infiltrative disease and biventricular hypertrophy (Fig. 1F–H).

Cardiac lymphoma is a rare clinical diagnosis with DLBCL accounting for 42% of all cases 1. By contrast, cardiac involvement is common in AL amyloidosis and occurs in about 50% of cases 2. Cardiac amyloidosis characteristically appears as diffuse patchy enhancement on DE‐CMR 3. Our case highlights that cardiac lymphoma may mimic amyloid infiltration of the myocardium on CMR, and is a particularly challenging diagnosis in the setting of transformed Waldenström's macroglobulinemia. Heightened clinical suspicion and early diagnosis of cardiac lymphoma using CMR is paramount as chemotherapy has been demonstrated to portent an increased survival rate 1.

## Authorship

LL, VM, IDCK, DSJ, and RK: contributed to the writing and approval of the final manuscript.

## Conflict of Interest

None declared.
